# Effects of Monochromatic Lighting During Incubation and Vaccination on the Splenic Transcriptome Profiles of Chicken

**DOI:** 10.3389/fgene.2021.628041

**Published:** 2021-05-20

**Authors:** Mohamed M. A. Ibrahim, Jill R. Nelson, Gregory S. Archer, Giridhar Athrey

**Affiliations:** ^1^Department of Laser Applications in Metrology, Photochemistry and Agriculture, National Institute of Laser Enhanced Sciences, Cairo University, Giza, Egypt; ^2^Department of Poultry Science, Texas A&M University, College Station, TX, United States; ^3^Faculty of Ecology and Evolutionary Biology, Texas A&M University, College Station, TX, United States

**Keywords:** monochromatic lighting, poultry, vaccination, Newcastle disease, transcriptome, spleen, incubation

## Abstract

Lighting is a crucial environmental variable in poultry operations, but illumination during incubation is relatively understudied. The ability to stimulate development or immune performance using *in ovo* lighting is a promising approach for improving poultry health and welfare. This study investigated how monochromatic green light during incubation and vaccination method and timing affected chicken splenic gene expression patterns. We performed this study with 1,728 Hy-Line white layer eggs incubated under two light treatments during incubation: continuous dark and continuous green monochromatic light, over the entire incubation period. Half the eggs in each light treatment received *in ovo* vaccination, applied on embryonic day 18 (ED18). The remaining half were vaccinated by spraying on hatch day. After hatching, the light treatments followed the industry-standard lighting regimens. The study had six treatment groups with light–dark pairs for non-vaccinated, *in ovo* vaccinated, and post-hatch vaccinated. We assessed splenic gene expression at ED18 and at 7 days post-hatch (PH) in all the treatments. We isolated and sequenced 24 mRNA libraries on the Illumina platform, followed by bioinformatics and differential gene expression analyses. RNAseq analysis showed between 62 and 6,755 differentially expressed genes (DEGs) between comparisons, with the most prominent differences observed between ED and PH samples, followed by comparisons between vaccination methods. In contrast, light vs. dark treatments at ED showed limited effects on transcriptomic profiles. However, we observed a synergistic effect of lighting during incubation on post-hatch vaccination responses, with differentially expressed genes (DEGs) unique to the light treatment showing stimulation of cell proliferation with significance for immune activity (inferred from gene ontology terms). Gene ontology and pathway analysis indicated biological processes like cellular component organization or biogenesis, rhythmic process, developmental process, response to stimulus, and immune system processes were explained by the DEGs. While lighting is an important source of circadian stimulation, other controlled studies are required to clarify whether *in ovo* circadian entrainment plays a role in modulating immune responses.

## Introduction

Over the past half century, global poultry production has increased over fivefold and continues to increase (FAO and OECD, 2016). Poultry is the world’s most consumed animal protein (OECD, 2018), and as a result of the increasing demand for poultry products, there is a growing emphasis on sustainable production. Morbidity and mortality rates, especially in early life, remain challenging, and approaches that improve health and performance are needed. One important approach to reduce morbidity is to improve egg incubation variables to produce healthy chicks that can cope with the housing environment and overcome potential infections.

Lighting is a ubiquitous management tool that plays a central role in poultry production, reproduction, and health. Light is a vital external abiotic cue for the chicken’s physiological development, behavior, health, and welfare (Parvin et al., 2014; Archer and Mench, 2017; Raccoursier et al., 2019). More recently, the role of lighting and photoperiods have become valuable tools for modulating the gut microbiota of poultry (Hieke et al., 2019; Parkar et al., 2019). These benefits have spurred intensive research into the use of monochromatic lighting to improve poultry health and production traits. Light-emitting diodes (LEDs), a monochromatic light source, are a promising approach to enhance avian productivity (Sultana et al., 2013; Zhang et al., 2016). Fowl, like other avian species, have exceptional sensitivity to different wavelengths of light and a wider visible range compared with humans owing to extra-retinal photoreceptors in the suprachiasmatic nucleus (SCN) and photoreceptors in the pineal gland (Csernus et al., 1999; Yamao et al., 1999; Withgott, 2000; Blackwell, 2002; Knott et al., 2010). The transduction of light to biological signals promotes physiological and growth performance (Foster and Follett, 1985; Rozenboim et al., 1999; Lewis and Morris, 2000; Mano and Fukada, 2007). In poultry, monochromatic lighting can reduce fear and stress responses and improve health and welfare (Sultana et al., 2013; Archer, 2017). Therefore, lighting during incubation is an emerging area of research for its influences on embryonic development, hatchability, and post-hatch chick health traits. The current industry practice is to incubate eggs under complete darkness.

Light exposure during development is beneficial; chick embryos can sense light early, which translates into positive effects on behavioral and physiological development (Rozenboim et al., 2004b; Halevy et al., 2006; Zhang et al., 2012). For example, lighting during incubation accelerates a chick’s embryogenesis and shortens incubation time compared with dark incubation (Shutze et al., 1962; Siegel et al., 1969; Adam and Dimond, 1971; Walter and Voitle, 1973). The use of a specific wavelength of light improve certain aspects like growth and hatchability characteristics and decrease the numbers of chicks with defects like unhealed navel, leg abnormalities, and other health defects (Cooper, 1972; Shafey, 2004; Archer, 2015, 2018; Archer et al., 2017). During incubation, light exposure may also modify hatching-linked hormones like thyroid T4, T3, and corticosterone, which may accelerate the time to hatch (Fairchild and Christensen, 2000; Shafey and Al-mohsen, 2002; Huth and Archer, 2015; Tong et al., 2018). Exposing chick embryos to warm and cool white LED lights showed a similar improvement effect on hatchability, chick quality, and decreased stress and fear responses post-hatch in broilers compared with conventional industrial dark incubation (S. Archer, 2016).

The benefits of *in ovo* lighting also translate into outcomes in the post-hatch environment. Blue light significantly increases muscle growth and satellite cell proliferation after 21 days post-hatch. The green spectrum is more effective in promoting muscle growth and satellite cell proliferation during post-hatch day 1 to day 21 (Cao et al., 2008; Liu et al., 2010). Red light reduces the proliferation of satellite cells, changes myofiber formation and muscle growth of broilers, and unlike green or blue light, upregulates insulin-like growth factor 1 receptors (IGF-1R) in skeletal muscle (Bai et al., 2016). Besides, welfare and behavioral traits are other variables influenced by the type of monochromatic light used. For example, broilers stay energetic and mobile under long wavelengths compared with short wavelengths, which might control tonic immobility in the fear response. These findings help the production and welfare parameters such as FCR, flock stress, and fear response (Sultana et al., 2013; Parvin et al., 2014; Archer and Mench, 2017).

Among these different monochromatic lights that had significant effects, the green spectrum shows many advantageous effects. Rozenboim et al. (2004a), Zhang et al. (2012) and Zhang L. et al. (2014) suggested that green light during embryogenesis improves the body and breast muscle weight during incubation and post-hatch by promoting satellite cell proliferation and differentiation in both late embryonic and newly hatched chicks. These experiments indicate that light stimulation during embryogenesis improves growth and productivity and long-term reductions in fearfulness (Chiandetti et al., 2013; Archer, 2017). Green light also promotes melatonin secretion from the chicks’ pineal gland during incubation (Jin et al., 2011; Jiang et al., 2016; Ma et al., 2018, 2019), and accelerates embryo development and modify hatch-related hormones, thyroid, and corticosterone resulting in earlier hatching (Tong et al., 2018). These hormonal interactions also suggest implications for immunity; green light stimulates proliferation of peripheral blood T- and B-lymphocytes, and the IL-2 concentration increased during the early stages post-hatch, which is crucial in the humoral response (Xie et al., 2008a,b; Sadrzadeh et al., 2011; Li et al., 2013; Zhang Z. et al., 2014). Taken together with the importance of lighting regimens for post-hatch gut health and performance, driven by circadian rhythms (Hieke et al., 2019; Wang et al., 2020), it is necessary to investigate how monochromatic lighting during incubation can improve health and production traits.

In this study, we investigated how green light biostimulation during incubation influences vaccination responses against Newcastle disease virus (NDV). We hypothesized that photostimulation during incubation would generate distinct responses to vaccination measured by splenic gene expression.

## Materials and Methods

### Animals and Experimental Design

We carried out all the live animal work using protocols approved by the Institutional Animal Care and Use Committee of Texas A&M University (AUP #2016-0051). We obtained White layer (*Gallus gallus domesticus*) fertilized eggs (*n* = 1,728) from a commercial hatchery (Hy-Line North America, LLC) and randomly distributed them among six treatments. We placed 288 eggs in each incubator (QFG 1550), with two incubators assigned for each treatment (Table 1).

**TABLE 1:** Summary of the six treatment groups in this study, showing the combination of monochromatic lights and vaccination strategies.

Treatment group	Light treatment	Vaccination method and age	Sampling age
Light not vaccinated (LNV)	Green monochromatic light	None	ED18
Dark not vaccinated (DNV)	Dark	None	ED18
Light *in ovo* vaccinated (LIV)	Green monochromatic light	*In ovo* (ED18)	PHD7
Dark *in ovo* vaccinated (DIV)	Dark	*In ovo* (ED18)	PHD7
Light post-hatch vaccinated (LPHV)	Green monochromatic light	Spray (PH1)	PHD7
Dark post-hatch vaccinated (DPHV)	Dark	Spray (PH1)	PHD7

*The main groupings were based on biostimulation during incubation and the method of vaccination. The experimental birds were administered vaccines either *in ovo* on embryonic day 18 (ED18) or day 1 post-hatch (Day 1). Vaccinated birds were sampled on post-hatch day 7 (PHD7).*

We fitted incubators with green monochromatic light-emitting (LED) panels, measured at an average of 515 nm before passing the white eggshell and an average of 517 nm after passing through the eggshell. In effect, the shell barrier did not noticeably alter the emitted spectrum, consistent with a previous report by Archer (2017). Incubators were illuminated with two vertical LED light bars at the backside of the egg trays and two on the incubator door (AgriShift^®^ TLP, Junglite Green^TM^ technology, Once^®^ Animal-Centric Lighting Systems), producing an average light intensity of 250 lx (measured at a total of nine locations) at the egg’s surface using a light meter (Extech 401 027, Extech Instruments, Nashua, NH, United States). In contrast, the irradiance was 0.8757 W/m^2^ (Everfine SFIM-3000, Hangzhou, China). Glass windows on the incubators were covered with opaque sheets to prevent light intrusion from outside. Incubators were illuminated for 24 h a day (LD 24:0) for the entirety of incubation in the light treatments. For the dark treatments (LD 0:24), incubators were not illuminated and were also outfitted with opaque window covers to prevent light intrusion during incubation.

We incubated eggs at standard conditions of 37.5°C and 55% relative humidity for 18 days. After 18 days, eggs were transferred to the hatchers and maintained at temperature and relative humidity levels of 36.9°C and 65%, respectively. The hatchers were not fitted with illumination sources as previous research has shown that circadian rhythms are established by day 18 when incubated with illumination (Archer, 2015). On embryonic day 18 (ED18), we administered the *in ovo* vaccination with Newcastle disease virus vaccine (INNOVAX^®^-ND-SB, Intervet) to the LIV and DIV treatments. Vaccines were administered via injection of a 1× dose in 100-μl volume into the amniotic fluid by a 1-inch 21G (0.819 mm outer diameter, OD) needle, preceded by puncturing the eggshell with an 18G needle (1.270 mm OD). The injection holes were sealed with food-safe grade clear silicone to prevent infection and dehydration. For the post-hatch vaccination groups (LPHV and DPHV), NDV vaccination (NEWHATCH^®^-C2, B1 Type, C2 Strain, Live Virus, Intervet) was administered on day 1 after hatch by spraying the chicks immediately before placement into rearing pens.

Regardless of the vaccination strategy, we checked all hatched chicks, and 100 healthy and active chicks were randomly selected and placed in floor pens (3.34 m^2^), equipped with tube feeders and nipple drinkers, and raised until 14 days of age. We followed the standard recommendation for lighting given in the Hy-Line management guide, providing 20:4 (Light: Dark) h of illumination in the first week (30–50 lux) followed by LD 19:5 h of lighting a day (25 lx) during the second week. Room temperatures were maintained at 32 ± 2°C for the first week and then decreased by 2–3°C for the second week.

### Sample Collection

For the LNV and DNV treatments, we collected the embryonic spleen (ED18). Ten eggs were randomly selected from each incubator with green light and the control (dark) treatment. Embryos were dissected immediately after breaking open the eggs. Spleen samples were excised and stabilized in 1:5 volume of RNA*later*^TM^ (Invitrogen, Carlsbad, CA, United States) and stored at 4°C moving to long-term storage at −80°C until RNA isolation. For sampling at post-hatch day 7, 10 chicks each were selected randomly from LIV, LPHV, DIV, and DPHV treatments (total 40 individuals) and were euthanized humanely using exposure to CO_2_, followed by cervical dislocation. We harvested tissues from euthanized chicks within 20 min postmortem, and the spleen samples were stored in the same way as described above. Euthanasia procedures were performed using protocols approved by the Texas A&M University’s Institutional Animal Care and Use Committee (IACUC AUP #2016-0051).

### RNA Isolation and Quantification

From each sample, approximately 15–30 mg of spleen tissue was homogenized in Trizol reagent (Invitrogen, Carlsbad, CA, United States) with 1 cm^3^ of 1.0 mm diameter ZIRCONIA beads (cat. no. 11079124zx) using a Mini-Beadbeater-96 (BioSpec, OK, United States). We extracted total RNA, followed by an initial quantitation using a NanoDrop^TM^ 1000 spectrophotometer (Thermo Fisher Scientific, MA, United States), and estimation of protein contamination (260/280 ratio) and other organic contamination (230/260 ratio). Samples of sufficient quality and quantity were checked further with a Bioanalyzer 2100 (Agilent Technologies, Inc., DE, United States) chip reader using Agilent RNA 6000 Nano kit (No: 5067-1511) to assess the whole sample RNA integrity number (RIN) and suitability for library preparation. We evaluated total RNA isolates with RIN 8.5 or higher for genomic DNA contamination with a Qubit^TM^ RNA BR assay, 20–1,000 ng/μl (Catalog number: Q10211) as well as Qubit^TM^ dsDNA BR assay, 100 pg/μl to 1,000 ng/μl (Catalog number: Q32853). Total RNA samples passing these quality checks were normalized by dilution at 400 ng/μl using nuclease-free water (NF water) and used for library preparation.

### RNA Library Preparation and Transcriptome Profile Generation

We used 200 ng of total RNA as input for library preparation following the QuantSeq 3′ mRNA-Seq Library Prep Kit FWD for Illumina kit protocol (Lexogen, Vienna, Austria). We used oligo (dT) primers and Illumina-specific Read-2 linker sequences to reverse transcribe mature (poly-A tailed) mRNA to produce a complementary first-strand DNA, followed by second-strand synthesis using a random primer containing the Illumina-specific Read-1 linker sequence in the presence of DNA polymerase enzyme. We cleaned libraries using a magnetic bead-based purification step to remove impurities that interfere with library enrichment and indexing steps. Enriched single-indexed libraries were cleaned and checked using the TapeStation 2200 system and the D1000 ScreenTape assay (Agilent Technologies, Inc.), and libraries were normalized to 4 nM. Twenty-four libraries (*N* = 4/treatment) were pooled in equimolar proportions and sequenced at the Texas A&M Institute for Genome Sciences and Society (TIGSS, College Station, TX, United States) on an Illumina NextSeq (Illumina, San Diego, CA, United States) platform. Libraries were sequenced in 75-bp single-end mode, generating an average of 8.8 million reads per library.

#### Transcriptome Data Analysis

We performed all bioinformatics analysis with open-source tools using well established RNAseq analysis pipelines. In summary, the single-end raw reads in FASTQ format were quality checked with FastQC (Babraham Institute, Cambridge, United Kingdom) version 0.11.9 and MultiQC version 1.9 (Martin, 2011; Ewels et al., 2016), followed by the removal of adapter contamination and Lexogen indices. We performed adapter removal and quality filtering (Phred Q > 30) and over 35 bp in length using Trim_Galore version 0.4.5 (Bolger et al., 2014). Reads passing quality filters were mapped to the *Gallus gallus* genome, Galgal6 (Version 6, Ensembl Release 99 GRCg6a, January 2020), using the *de novo* splice mapper STAR (version STAR_2.5.3a_modified) (Dobin et al., 2013; Dobin and Gingeras, 2015). We counted the single-end reads mapped to exon features using HTSeq-count (version 0.9.1) (Anders et al., 2015).

We analyzed differential gene expression based on read counts using the package EdgeR (version 3.26.8) in the R statistical platform (version 3.6.2) (Robinson et al., 2010; McCarthy et al., 2012) using a two-factor model. Genes with uniformly low expression (<1 CPM) were not included in further analysis. We applied normalization factors to correct for differences in library sizes and estimated common and tagwise dispersion (generalized linear model). We performed DGE analysis with a two-factor design. The GLM approach is better at handling factorial designs with the interaction of photo-biostimulation and vaccination method, so we used the likelihood ratio test “glmLRT” function to test for significant differential expression between groups at FDR < 0.05. We applied the “glmLRT” test for all comparisons except three comparisons where we tested only the effect of monochromatic light (single factor). For the comparisons, LNV vs. DNV, LIV vs. DIV, and LPHV vs. DPHV, we ran the single factor analysis with the Exact test “decideTestsDGE.” We performed a power analysis based on the common dispersion (0.069) in our RNAseq data using SSizeRNA 1.3.2 (Bi and Liu, 2016), which showed that our design had 97% power to detect log_2_-fold differences of 2 at FDR ≤ 0.05.

### Pathway Analyses

Differentially expressed genes were subjected to further analysis using the Ingenuity Pathway Analysis (IPA; QIAGEN Inc.) software (Krämer et al., 2014) to reveal canonical pathways, networks activated by these DEGs, and their roles in molecular and cellular functions and physiological system development and function.

## Results and Discussion

### RNA Sequence Results and Identification of Differentially Expressed Genes

Sequencing of the 24 RNAseq libraries, with four biological replicates per treatment group (six treatments), generated a total of 211.2 million reads, with an average of 8.8 million reads per library. After quality filtering and adapter trimming, we retained 96.34–97.49% of the reads per replicate (Supplementary Table 1). An average of 93.71–95.05% mapped (Supplementary Table 2) to the genome reference (Galgal6, ENSEMBL 99 released in January 2020), and an average of 58% of the reads mapped uniquely to exons using HTSeq-Count (Supplementary Table 3). The common dispersion estimate for the entire dataset was low (0.069). Tagwise dispersion values in the dataset indicated that 75% of genes had a biological coefficient variation (BCV) below 0.11. A total of 24,356 genes were detected, of which 12,769 genes were expressed at CPM > 1. Of these, 11,300 genes were annotated on ENSEMBL, while the rest were novel transcripts with no annotations. Most (91%) of the expressed genes were protein coding, and the rest were assigned to long non-coding RNAs (lncRNAs, 7%), pseudogenes (0.8%), small nucleolar RNAs (snoRNAs, 0.49%), microRNA (miRNA, 0.25%), mitochondrial transfer RNA (Mt-tRNA, 0.11%), and small nuclear RNA (snRNA, 0.78%).

The analysis of differential expression using EdgeR showed that the fraction of DE genes ranged from 0.5 to 55%. The highest numbers of differentially expressed genes (FDR < 0.05) were in the comparison of groups differing in the method of vaccination (*in ovo* vs. post-hatch). The effect of green light biostimulation during incubation showed fewer, but noteworthy differences. The post-hatch comparisons revealed the fewest differences. All comparisons made are presented in Supplementary Table 4.

### Incubation With Monochromatic Green Light Stimulates Gene Expression Important for Immune Response and Energy Metabolism in the Embryonic Spleen

**FIGURE 1:**
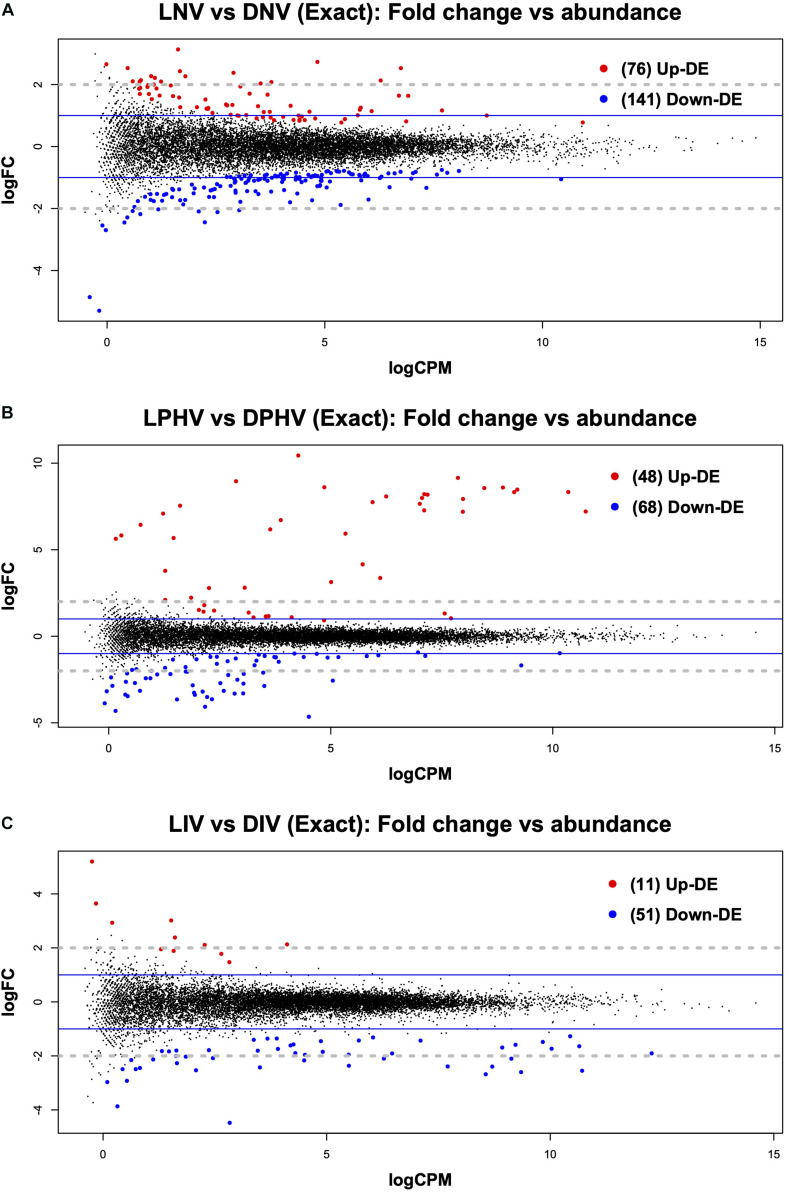
Mean average (MA) plot highlighting the log fold change and average abundance of each gene in pre- vs. post-hatch spleen transcriptome comparison. Significantly up and down DE genes and their counts are highlighted in red and blue, respectively. **(A)** Shows the DEGs in the embryonic spleen (E18) during green monochromatic light biostimulation pre-hatch in LNV vs. DNV treatment groups. **(B)** Shows the DEGs in spleen tissue post hatch (D7) in LPHV vs. DPHV treatment groups that received vaccination post hatch on day one. **(C)** Shows the DEGs in spleen tissue post hatch (D7) in LIV vs. DIV treatment group received *in ovo* vaccination E18. The *Y*-axis corresponds to the mean average of log10 count per million (CPM), and the *X*-axis displays the log2 FC, with significance assessed at FDR < 0.05. LNV, light not vaccinated; DNV, dark not vaccinated; LPHV, light post-hatch vaccinated; DPHV, dark post-hatch vaccinated; LIV, light *in ovo* vaccinated; DIV, dark *in ovo* vaccinated.

In the embryonic spleen (ED18), we saw 217 genes differentially expressed (FDR < 0.05) between the LNV and DNV treatments. Of these, 76 were upregulated in LNV, and 141 were downregulated (Figure 1A). Analysis of the enriched gene ontology (GO) terms with the DAVID database (using Entrez gene IDs against the chicken reference) returned 197 genes classified into biological process (BP), cellular component (CC), and molecular function (MF). The top enriched biological process terms were “Plasminogen Activation,” “Positive Regulation of Protein Secretion,” and “Cell-Matrix Adhesion,” for cellular components, the top enriched terms included “Blood Microparticles,” “Fibrinogen Complex,” and “Extracellular Exosome,” whereas the top enriched molecular functions were “Metallocarboxypeptidase Activity,” “Small Molecule Binding,” and “Oxygen Binding” (Supplementary Table 4). All three GO categories are presented in Supplementary Table 5.

Ingenuity pathway analysis (IPA) classified the 217 DEGs to 184 molecules annotated in its database. These molecules participate in 97 canonical pathways, of which 29 canonical pathways had a significant Z-score weighting. The top three activated pathways (based on Z-score) were “NF-κB Signaling,” “Sirtuin Signaling Pathway,” and “Sumoylation Pathway.” The top three inhibited pathways included “SPINK1 Pancreatic Cancer Pathway,” “LXR/RXR Activation,” and “NER Pathway” (Supplementary Table 5, LNV vs. DNV canonical Pathways). Nuclear factor kappa-light-chain-enhancer of activated B-cell signaling pathway (NF-κB), a protein complex that controls transcription of DNA (Liu et al., 2017), cytokine production, and cell survival was the top activated pathway with green light incubation, involving five genes, three (*INS*, *IRAK4*, and *PIK3CG*) of which were upregulated. Two (*HDAC1* and *NFKBIA*) were downregulated.

Finding the NF-κB and the Sirtuin-signaling pathways activated in this comparison is notable. The circadian core oscillator gene CLOCK controls the transcription factor NF-κB (Spengler et al., 2012), and in turn, the NF-κB pathway, which includes over 100 genes, regulates a variety of biological responses, particularly related to immune responses and inflammation (Dolcet et al., 2005). Similarly, sirtuins, which influences processes such as inflammation, stress response, and aging are known regulators of circadian transcription (Chang and Guarente, 2013; Masri et al., 2015). These associations between the circadian oscillator and the immune-response pathways suggests that photostimulation during development invokes this coupled mechanism’s activation, which is promising in terms of the applicability of monochromatic lighting during incubation.

### Green Monochromatic Light Stimulates Innate Immune Activity Following Post-hatch Vaccination

The post-hatch samples showed relatively fewer differentially expressed genes in the spleen irrespective of the vaccination method (*in ovo* or post-hatch). LPHV vs. DPHV groups revealed 116 DEGs at FDR < 0.05, where 48 were upregulated, and 68 were downregulated (Figure 1B). The enriched GO terms were based on 107 genes annotated in DAVID. The top three enriched biological process terms were “plasminogen activation,” “positive regulation of heterotypic cell-cell adhesion,” and “protein polymerization.” The top three cellular component terms were “blood microparticle,” “extracellular space,” and “extracellular exosome,” whereas the top molecular function terms were “metallocarboxypeptidase activity,” “small molecule binding,” and “hormone activity” (Supplementary Table 4).

Based on the 116 DEGs in LPHV vs. DPHV groups, IPA classified 88 to annotated molecules (39 molecules upregulated and 49 downregulated). These molecules were part of 35 canonical pathways, of which six were significant based on activation Z-score. The “Acute Phase Response Signaling” (APR) pathway (Venteclef et al., 2011) was predicted to be activated. In contrast, five pathways were predicted to be inhibited, namely, “LXR/RXR Activation,” “SPINK1, Pancreatic Cancer Pathway,” “Production of Nitric Oxide and Reactive Oxygen Species in Macrophages,” “Coagulation System,” and “Intrinsic Prothrombin Activation Pathway” (Supplementary Table 5, LPHV vs. DPHV Canonical Pathways). The activated APR pathway which has a role in the rapid inflammatory response (Castell et al., 1989; Gruys et al., 2005; Cray et al., 2009; Janciauskiene, 2013) included 12 DEGs, three of which were upregulated (*FGA*, *FGB*, and *FGG*) and four of which were downregulated (*ALB*, *AMBP*, *APOH*, and *TRR*).

The APR pathway is involved in various early defense against various stressors, of which chief among them are innate immune responses (Cray et al., 2009). One of the modalities is invoking a local proinflammatory cytokine response, which subsequently triggers downstream processes such as protease inhibition, clotting, and opsonization (Koj, 1996; O’Brien, 2012). The activation of this innate immune activity is further supported by our finding of the GO terms for “plasminogen activation” and “blood microparticles.” Both these suggest the associated inflammation and coagulation responses, where coagulation proteases may modulate the inflammatory response. The activated pathways show that biostimulation during incubation enhanced these immune responses to vaccination. The interleukins IL-22 and IL-6 are both regulators of the APR protein synthesis (Castell et al., 1989; Liang et al., 2010). These genes have strong diurnal oscillations corresponding to circadian expression (Nilsonne et al., 2016). While we did not find significant differences in CLOCK gene expression in this comparison, the activation of the APR pathway, which is under circadian control, suggests a role for stimulation of circadian-regulated processes. These results indicate that the incipient circadian system was stimulated in embryos exposed to monochromatic green light, which in turn appears to stimulate specific innate immune responses.

The *in ovo* vaccinated groups (LIV vs. DIV) showed few (62 DEGs at FDR < 0.05) differences at day 7 post-hatch, but this is not surprising given that lighting was the only variable (Figure 1C). The top three biological processes were “lipid catabolic process,” “transport,” and “positive regulation of ERK1 and ERK2 cascade,” the top three cellular components were “extracellular region,” “extracellular space,” and “blood microparticles,” whereas molecular functions had only two enriched GO terms “metallocarboxypeptidase activity” and “fatty acid-binding” (Supplementary Table 4).

Ingenuity pathway analysis classified 46 of the DEGs to annotated molecules, contributing to 19 canonical pathways, of which only one pathway was activated based on Z-score, the “serine protease inhibitor Kazal-type 1” (SPINK1) pathway (Supplementary Table 5, LIV vs. DIV canonical pathway). SPINK1 is a protein that cleaves prematurely activated trypsin to prevent the enzyme from causing cellular damage to the organ. All nine genes in this pathway were downregulated (*CELA1*, *CLPS*, *CPA1*, *CPA2*, *CPA5*, *CPB1*, *CTRB2*, *CTRC*, and *CTRL*). The SPINK1 secretory protein is protective of pancreatic function but can also be active in promoting tumor progression (Mehner and Radisky, 2019). In this case, as no pathologies are involved, the activation of this pathway suggests the former activity (protective). In some cancers, the SPINK1 protein modulates cancer cells’ tolerance, regulating apoptosis, and maintaining the body’s natural immune surveillance system (Lu et al., 2011). Therefore, the involvement of cell-mediated immunity in the context of vaccination in this study is noteworthy. The efficacy of vaccines is dependent on both a humoral and cell-mediated immune response (Amanna and Slifka, 2011). Previous studies of NDV vaccination response have noted that cell-mediated immunity is important in decreasing disease and transmission potential (Kapczynski et al., 2013). While we would expect that both the LIV and DIV groups would elicit the same immune responses, factors that improve these responses would be highly relevant from an application standpoint. If embryonic stimulation with the green light is indeed better at stimulating the cell-mediated immune component of vaccine response, as suggested by our results, this is an important outcome of our work. The observation of SPINK1 activation in the *in ovo* vaccinated, but not the post-hatch vaccinated group, is another notable difference. It remains to be determined if the post-hatch environment (absence of green monochromatic lighting) contributed to this observation.

### Similarity of Expression Networks

We assessed gene co-expression networks to characterize correlations and directionality of expression. We identified 13 networks for the LNV vs. DNV comparison, and six networks were identified for both LPHV vs. DPHV and the LIV vs. DIV comparisons. The networks identified from the DEGs in spleen samples pre- and post-hatch by IPA are presented in Supplementary Table 6.

**FIGURE 2:**
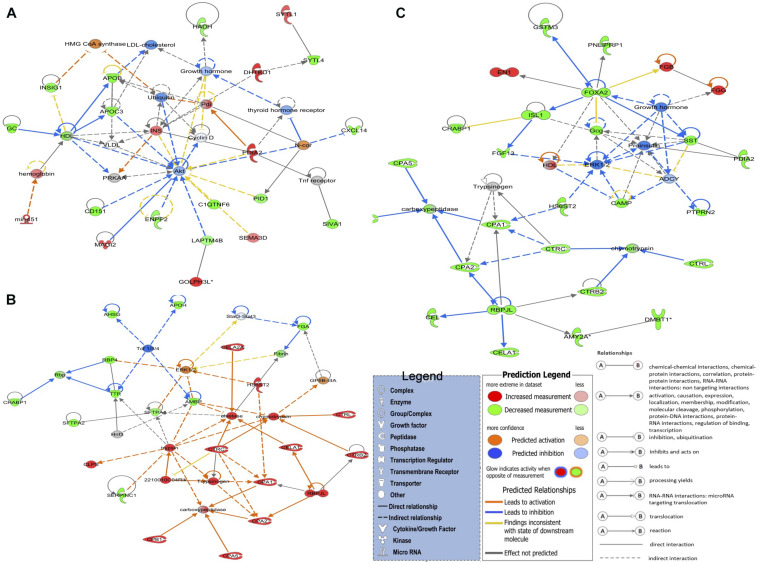
The top three detected gene networks underline the affected genes in green monochromatic light biostimulation comparisons during incubation and their interaction in potentially regulating developmental biological processes pre- and post-hatch generated by QIAGEN’s Ingenuity Pathway Analysis (IPA, QIAGEN Inc.) (Krämer et al., 2014). **(A)** LNV vs. DNV gene network for cell morphology, digestive system development and function, and organ morphology. **(B)** LPHV vs. DPHV gene network for carbohydrate metabolism, lipid metabolism, and protein synthesis. **(C)** LIV vs. DIV gene network for developmental disorder, hematological disease, and hereditary disorder. Differentially expressed genes in the biostimulated comparisons were used in the ingenuity pathway analysis, and significant gene networks based on IPA scores were identified. Genes highlighted in red were upregulated, while those highlighted in green were downregulated in all biostimulated comparisons.

The top networks (Figure 2A) in the LNV vs. DNV comparison include 21 genes involved in cell morphology, digestive system development and function, and organ morphology. The genes that were upregulated in embryos stimulated by green light were *DHTKD1*, *GOLPH3L*, *INS*, *MAGI2*, *mir-451*, *PDIA2*, *SEMA3D*, and *SYTL1*, while the downregulated genes were *APOB*, *APOC3*, *C1QTNF6*, *CD151*, *CXCL14*, *ENPP2*, *GC*, *HADH*, *INSIG1*, *LAPTM4B*, *PID1*, *IVA1*, and *SYTL4*. Notably, in this network, the apolipoprotein transporters (APOB and APOC3) were downregulated, while insulin was upregulated. The reciprocity between insulin and lipoproteins is reported from various insulin resistance disorders (Duivenvoorden et al., 2005; Haas et al., 2013; Åvall et al., 2015), but what it means, besides metabolic signaling, is not clear. This interpretation is also supported by the GO term and pathway analyses.

The top network in LPHV vs. DPHV comparison (Figure 2B) contained 23 genes involved in carbohydrate metabolism, lipid metabolism, and protein synthesis biological function. The network includes the genes *2210010C04Rik*, *CELA1*, *CELA2A*, *CLPS*, *CPA1*, *CPA2*, *CPA5*, *CPB1*, *CTRB2*, *CTRC*, *CTRL*, *HS6ST2*, and *RBPJL*, which were upregulated in spleen samples from chicks vaccinated post-hatch, and the genes *AHSG*, *AMBP*, *APOH*, *CRABP1*, *FGA*, *RBP4*, *SERPINC1*, *SFTPA1*, *SFTPA2*, and *TTR* were downregulated. The upregulation of several chymotrypsin (CEL) genes is indicative of pancreatic activity involved in the breakdown of proteins, indicating a more pronounced enzymatic activity associated with metabolism. As the circadian is a regulator of metabolic signaling, these observations again indicate a beneficial role of biostimulation on metabolic performance.

The top network in LIV and DIV group (Figure 2C) showed 27 genes that are associated with developmental disorder, hematological disease, and hereditary disorder (GO terms). Genes *EN1*, *FGB*, and *FGG* were upregulated in spleen samples from photostimulated chicks that received *in ovo* vaccination, while *AMY2A*, *CAMP*, *CEL*, *CELA1*, *CPA1*, *CPA2*, *CPA5*, *CPB1*, *CRABP1*, *CTRB2*, *CTRC*, *CTRL*, *DMBT1*, *FGF13*, *FOXA2*, *Gcg*, *GSTM3*, *HS6ST2*, *ISL1*, *PDIA2*, *PNLIPRP1*, *PTPRN2*, *RBPJL*, and *SST* genes were downregulated.

### Overrepresented Gene Ontology and Pathway Terms Indicate Lighting Stimulates Early Life Metabolic Activity

**FIGURE 3:**
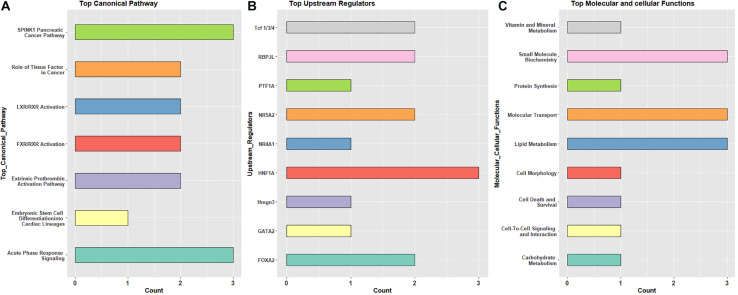
The frequently observed pathway terms based on differentially expressed genes in pre- and post-hatch birds incubated in green monochromatic light versus dark. **(A)** top canonical pathway, **(B)** upstream regulator, **(C)** top molecular and cellular functions. All DEGs from pre (E18) and post-hatch (D7) were subjected to IPA analysis to detect molecules across observations.

Specific canonical pathways (from IPA analysis) were observed repeatedly across the three pairwise comparisons. Shared pathway terms from different comparisons indicate metabolic and immune functions, as well as transcriptional activity controlling developmental processes. Similarly, there were recurring overlaps in the list of upstream regulators, molecular and cellular functions, and physiological system development and function (Supplementary Table 4). These terms are summarized in Figure 3. The most observed canonical pathways are serine protease inhibitor Kazal type 1 (*SPINK1*) and acute phase response signaling (Figure 3A), while the most observed upstream regulators include *HNF1A*, *FOXA2*, and *NAR5A2* (Figure 3B). These transcription factors are expressed in several tissues and are known to be important in development and, in this case, do not provide additional context to the canonical pathways. Moreover, the most observed molecular and cellular functions include lipid metabolism, molecular transport, and small-molecule biochemistry (Figure 3C).

**FIGURE 4:**
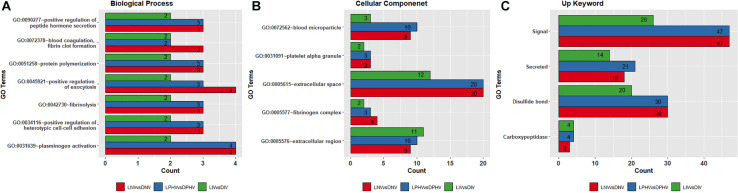
Overlapped Gene Ontology enrichment terms based on DEGs in pre- and post-hatch from illuminated and dark treatments. **(A)** biological process, **(B)** cellular component, and **(C)** Up_Keywords. All DEGs from pre (E18) and post-hatch (D7) were subjected to the DAVID database for Gene Ontology (GO) enrichment analysis. All the GO terms were significant based on a modified Fisher Exact *P*-value < 0.05.

Shared GO terms all indicate ongoing developmental processes, and several overrepresented terms (shown in Figure 4). Biological process terms were repeated across the three pre- and post-hatch comparisons indicating background molecular events including regulation of blood coagulation and vessel formation, different types of cell-to-cell attachment, protein–polymer formation, and regulation of hormone-based protein secretion (Figure 4A and Supplementary Table 4). Blood coagulation, fibrin clot formation (GO:0072378), plasminogen activation (GO:0031639), and fibrinolysis (GO:0042730) all invoke the coagulation system, and angiogenesis in wound healing. Positive regulation of exocytosis (GO:0045921), positive regulation of heterotypic cell–cell adhesion (GO:0034116), positive regulation of peptide hormone secretion (GO:0090277), and protein polymerization (GO:0051258) indicate biogenesis, regulation of hormone levels, and heterotypic cell–cell adhesion. Biological process GO terms detected in only two of the biostimulation comparisons, and unique ones are presented in Supplementary Table 5.

Biomolecules that have specific functions in plasma membranes or adjacent areas were the most enriched and overrepresented cellular component GO terms across comparisons (Figure 4B and Supplementary Table 5). These GO terms include blood microparticle (GO:0072562), extracellular region (GO:0005576), extracellular space (GO:0005615), fibrinogen complex (GO:0005577), and platelet alpha granule (GO:0031091), which play a role in the organization of protein microparticles and gene products secreted from a cell but retained within the organism (i.e., released into the interstitial fluid or blood). GO terms found only in the LIV and DIV treatment showed a role in lipid metabolism (GO:0060417), chylomicron (GO:0042627), high-density lipoprotein particle (GO:0034364), and very low-density lipoprotein particle (GO:0034361) (Supplementary Table 5). In addition, the overlapping Up_Keywords included: “Signal,” “Secreted,” “Disulfide bond,” and “Carboxypeptidase,” indicating molecular and cellular functions broadly involved in catalysis and cell signaling (Figure 4C and Supplementary Table 5).

We analyzed the pathways enriched for the differentially expressed genes on ED18 and post-hatch treatments on day 7 using the Kyoto Encyclopedia of Genes and Genomes (KEGG) (Kanehisa et al., 2016). KEGG pathways were considered enriched if at least two DEGs were found in the background pathway and a modified Fisher exact *P*-value < 0.05. In the embryonic spleen (LNV vs. DNV), three KEGG pathways were significantly enriched, including oxidative phosphorylation followed by the Toll-like receptor signaling pathway and RNA polymerase. In the post-hatch spleen, metabolic pathways and PPAR signaling pathways were enriched in LPHV vs. DPHV, whereas metabolic pathways were the only enriched pathway in LIV vs. DIV. KEGG IDs for each comparison, along with fold enrichment value and incorporated Entrez ID genes, are presented in Table 2.

**TABLE 2:** The Kyoto Encyclopedia of Genes and Genomes (KEGG) pathway enrichment analysis of differentially expressed genes in spleen tissues on ED18 of incubation, LNV and DNV treatments, and day 7 post-hatch, LPHV, DPHV, LIV, and DIV treatments, that were exposed to green monochromatic light during incubation, were subjected to the DAVID database for pathway enrichment analysis.

Group	KEGG ID	Pathway terms	Fold enrichment	Entrez IDs of genes
LNV vs. DNV	gga00190 gga04620 gga03020	-Oxidative phosphorylation -Toll-like receptor signaling pathway -RNA polymerase	3.05 2.5 1.5	-PPA2, NDUFA5, NDUFS6, COX7C, UQCRFS1, NDUFB5 -NFKBIA, PIK3CG, TOLLIP, MAPK12, IRAK4 -POLR1D, POLR2F, POLR2D
LPHV vs. DPHV	gga01100 gga03320	-Metabolic pathways -PPAR signaling pathway	14.3 2.9	-PNLIPRP1, PSPH, ALDOB, SIIL, PLA2G1B, ATP5A1W, CEL, FUT9, ADH1C, GATM, TCIRG1, HPD, PLA2G1BL, AMY2A, UGT1A1 -SCD, APOC3, FABP1
LIV vs. DIV	gga01100	-Metabolic pathways	12.5	-PNLIPRP1, SIIL, PLA2G1B, CEL, PLA2G1BL, FUT9, AMY2A

*All the pathways with a modified Fisher exact *P*-value < 0.05 and a threshold gene count of 2 were considered enriched.*

**FIGURE 5:**
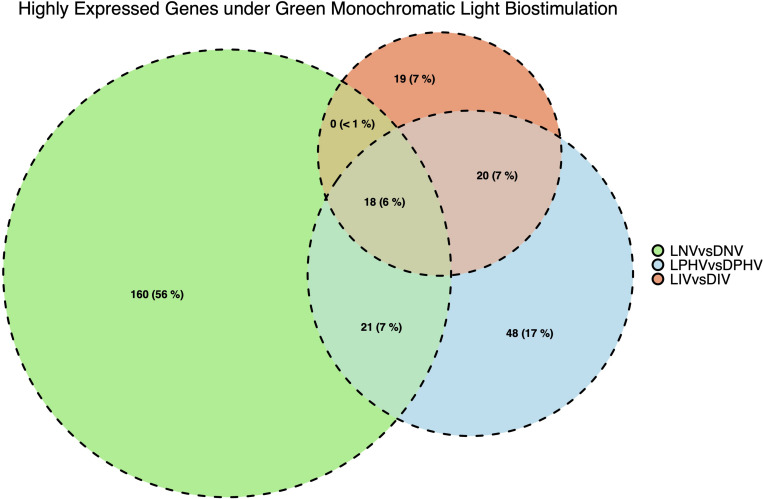
Number and overlapping DEGs in spleen tissues between pre-hatch (LNV vs. DNV) and post-hatch (LPHV vs. DPHV and LIV vs. DIV) treatments, stimulated by green monochromatic light during incubation. The DEGs were determined by statistical algorithms EdgeR. Notably, embryonic spleen samples had a greater number of highly expressed DEGs (FDR < 0.05) compared to post-hatch spleen samples indicating the dilution of biostimulation in the post-hatch environment (shift to standard lighting).

**FIGURE 6:**
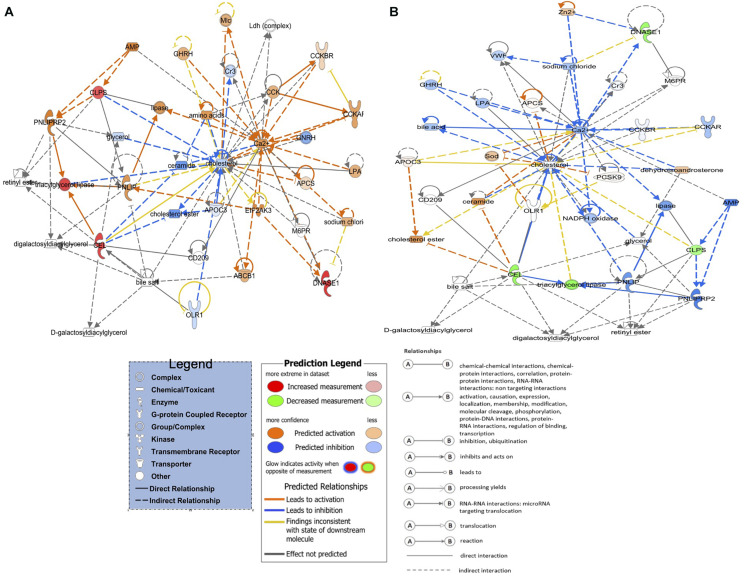
Activated and inhibited lipid metabolism networks in the green light biostimulation comparisons underline the role of green monochromatic light biostimulation regulating developmental biological processes pre- and post-hatch. **(A)** LNV vs. DNV and LPHV vs. DPHV gene network activated lipid metabolism. **(B)** LIV vs. DIV gene network for inhibited lipid metabolism. Genes highlighted in red were upregulated, while those highlighted in green were downregulated in all biostimulated comparisons.

Eighteen DEGs were found to be overlapping in green monochromatic light biostimulation treatments in comparison with full dark groups during incubation (Table 3). The overlap between the total DEGs (217, 116, and 62) that were detected in LNV vs. DNV, LPHV vs. DPHV, and LIV vs. DIV comparisons are shown in Figure 5. We analyzed the 18 shared DEGs using IPA, of which 15 genes were mapped to known orthologs. Seven genes, *Colipase*, *Carboxypeptidase A1*, *Carboxypeptidase A5*, *Carboxypeptidase B1*, *Chymotrypsinogen B2*, *Chymotrypsin C*, and *Chymotrypsin like*, were involved in one canonical pathway “SPINK1” that was inhibited in the LNV vs. DNV group, and the LPHV vs. DPHV group, while it was activated in the LIV vs. DIV group. The networks identified from the 18 shared DEGs are presented in Table 3. IPA network analysis showed an overrepresented network with lipid metabolism, molecular transport, and small-molecule biochemistry functions in the three comparisons. Three genes *CEL*, *CLPS*, *DNASE1*, and *triacylglycerol lipase*, were upregulated in the LNV vs. DNV group, and LPHV vs. DPHV (Figure 6A), unlike the LIV vs. DIV group where those genes were downregulated (Figure 6B). The genes in this network encode for proteins involved in lipid metabolism, e.g., carboxyl ester lipase (CEL) secreted from the pancreas to breakdown cholesterol and lipid-soluble vitamin ester hydrolysis and absorption, and protein metabolism. Also, carboxypeptidase, which hydrolyzes a C-terminal peptide bond in polypeptide chains, signal proteins involved in cell differentiation, proliferation, and photoreceptor proteins that convert the light waves into signals, e.g., light-absorbing chromophores (Karu, 1996; Shichida and Matsuyama, 2009; El-Gendy et al., 2015; de Freitas and Hamblin, 2016; Losi et al., 2018). The activity in pancreatic tissue is mostly upregulated, which is consistent with the need for enzymes to metabolize the fat and protein from egg yolk to support the embryogenesis process. This is consistent with the finding of Zhang et al. (2016), where they reported that the embryos in the green light group developed faster, resulting in higher nutrient consumption from the yolk, showing a lower weight percentage of yolk retention on ED19 of embryogenesis and a day after hatching.

**TABLE 3:** Gene networks from the 18 shared differentially expressed genes for monochromatic green light biostimulation groups converted to human orthologous genes.

Gene name	Gene description	LNV vs. DNV LogFC	LPHV vs. DPHV LogFC	LIV vs. DIV LogFC
FGB	Fibrinogen beta chain [373926]	–1.63	–3.65	1.89
HS6ST2	Heparan sulfate 6-O-sulfotransferase 2 [395150]	2.08	2.23	–1.57
CPA1	carboxypeptidase A1 [395276]	2.73	8.47	–1.73
LBFABP	Liver basic fatty acid-binding protein [395345]	–2.45	–3.31	1.95
PIT54	PIT54 protein [395364]	–2.08	–4.07	2.11
DNASE1	Deoxyribonuclease 1 [395725]	2.21	8.96	–1.90
FGG	Fibrinogen gamma chain [395837]	–1.76	–3.26	2.38
ALB	Albumin [396197]	–1.88	–4.65	2.13
SST	Somatostatin [396279]	1.64	7.75	–1.43
CPA5	Carboxypeptidase A5 [416683]	2.27	7.65	–2.40
CEL	Carboxyl ester lipase [417165]	1.97	8.21	–2.10
CPB1	Carboxypeptidase B1 [424888]	1.58	9.15	–1.59
CTRL	Chymotrypsin like [427531]	1.87	7.27	–2.68
CTRC	Chymotrypsin C [430670]	2.53	8.33	–1.27
CTRB2	Chymotrypsinogen B2 [431235]	2.10	8.18	–2.39
CLPS	Colipase [771102]	1.67	7.19	–1.48
PDIA2	Protein disulfide isomerase family A member 2 [100857897]	2.27	5.93	–2.36
LOC101749216	Uncharacterized LOC101749216 [101749216]	3.13	10.44	–1.96

### Limited but Notable Interaction of Biostimulation and Vaccination

**FIGURE 7:**
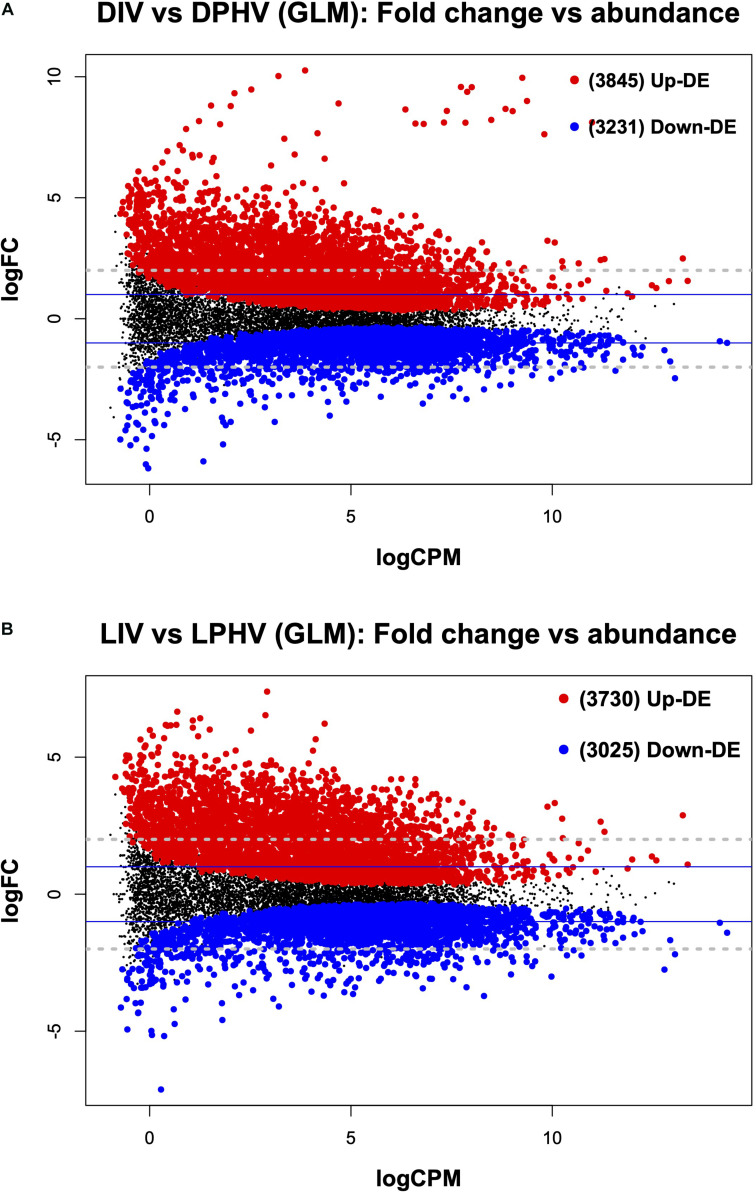
The mean average (MA) plot highlighting the log fold change and average abundance of each gene, comparing the differences between the method of vaccination with or without green monochromatic light on the spleen transcriptomes collected on day 7 post-hatch. Significantly up and down DE genes and their numbers are highlighted in red and blue, respectively. **(A)** Shows the DEGs in the post hatch spleen tissue (D7) receiving either *in ovo* or post-hatch vaccination in the unilluminated treatment. **(B)** Shows the DEGs in the post-hatch spleen tissue (D7) receiving either *in ovo* or post-hatch vaccination in green light treatments. The *y*-axis corresponds to the mean average of log counts per million (CPM), and the *x*-axis displays the log2FC. DIV, dark *in ovo* vaccinated; LIV, light *in ovo* vaccinated; DPHV, dark post-hatch vaccinated; LPHV, light post-hatch vaccinated.

It is important to note that *in ovo* vaccination with NDV is associated with a delay in chick hatching (Dimitrov et al., 2017). Here, we found that the method of vaccination (*in ovo* vs. spraying) altered the transcriptomic profile in spleen tissues post-hatch regardless of lighting during incubation. In the DIV vs. DPHV comparison, we saw 7,076 differentially expressed genes (FDR < 0.05). Of these, 3,845 were upregulated in DIV, and 3,231 were downregulated (Figure 7A). Of the 1,432 enhanced DEGs queried against the DAVID database, 347 genes were enriched for 51 biological processes. The top BP terms were “positive Regulation of Transcription from RNA Polymerase II Promoter” and “Regulation of Rho Protein Signal Transduction,” and “Heart Development.” Nineteen cellular component terms were enriched based on 545 incorporated genes, with the top terms including “Nucleoplasm,” “Kinesin Complex,” and “Nucleus.” Twenty-six molecular functions were enriched, among which, the top MF terms were “metal ion binding,” “zinc ion binding,” and “ATP binding.” The top GO terms are presented in Supplementary Table 4. KEGG analysis showed 166 upregulated genes enriched in 21 pathways, the top three of which were “MAPK signaling pathway,” “Insulin resistance,” and “Regulation of actin cytoskeleton” (Supplementary Table 4).

From the DEGs between DIV vs. DPHV, 1,556 genes were enhanced at LogFC greater than 2, with 197 downregulated and 1,359 upregulated. In IPA, these 1,556 genes were part of 263 canonical pathways, of which 235 were predicted to be activated, and 23 pathways were predicted to be inhibited based on Z-score. The top five significant canonical pathways include “Protein Kinase A Signaling,” “Cardiac Hypertrophy Signaling (Enhanced),” “Factors Promoting Cardiogenesis in Vertebrates,” “SAPK/JNK Signaling,” and “Synaptogenesis Signaling Pathway” (Supplementary Table 4). The top five canonical pathways predicted to be activated were “Cardiac Hypertrophy Signaling (Enhanced),” “Superpathway of Inositol Phosphate Compounds,” “Cardiac Hypertrophy Signaling,” “3-phosphoinositide Biosynthesis,” and “NF-κB Signaling,” while the top five canonical pathways predicted to be inhibited are “Pancreatic Cancer Pathway (SPINK1),” “LXR/RXR Activation,” “PTEN Signaling,” “Endocannabinoid Cancer Inhibition Pathway,” and “RhoGDI Signaling”; full weighted canonical pathway-based Z-score are presented in Supplementary Table 6.

In the LIV vs. LPHV comparison, we found a total of 6,755 differentially expressed genes (FDR < 0.05), of which 3,730 were upregulated in LIV (Figure 7B). DEGs were queried against the DAVID database, where the top enriched terms were “Microtubule-Based Movement,” “Cell Migration,” and “Positive Regulation of Transcription from RNA Polymerase II Promoter.” We saw 23 cellular component GO terms enriched, and the top three terms were “nucleoplasm,” “kinesin complex,” and “cytoplasm.” Genes (456) involved in the enrichment of molecular functions yielded the top molecular functions “zinc ion binding,” “ATP binding,” and “metal ion binding.” Upregulated genes (112) were enriched in 13 KEGG pathways, with the top pathways being “Fanconi Anemia Pathway,” “MAPK Signaling Pathway,” and “Endocytosis” (Supplementary Table 4). Complete GO terms, which are results of the DAVID annotation tool, are listed in Supplementary Table 6.

Of the enhanced DEGs (LogFC > | 2|), 1,476 genes were annotated in IPA (1,266 upregulated). Those molecules were enriched in 252 canonical pathways, of which 227 pathways were predicted to be activated, 18 pathways were predicted to be inhibited based on the weighted Z-score, and the rest could not be predicted due to zero Z-core values. The most significant five canonical pathways encompass “Protein Kinase A Signaling,” “B Cell Receptor Signaling,” “Cardiac Hypertrophy Signaling (Enhanced),” “Superpathway of Inositol Phosphate Compounds,” and “SAPK/JNK Signaling” (Supplementary Table 4). The top five activated pathways (based on Z-score) included “Cardiac Hypertrophy Signaling (Enhanced),” “Superpathway of Inositol Phosphate Compounds,” “3-phosphoinositide Biosynthesis,” “3-phosphoinositide Degradation,” and “D-myo-inositol-5-phosphate Metabolism.” The top five inhibited pathways included “PTEN Signaling,” “Endocannabinoid Cancer Inhibition Pathway,” “PPAR Signaling,” “RhoGDI Signaling,” and “VDR/RXR Activation” (Supplementary Table 6).

**FIGURE 8:**
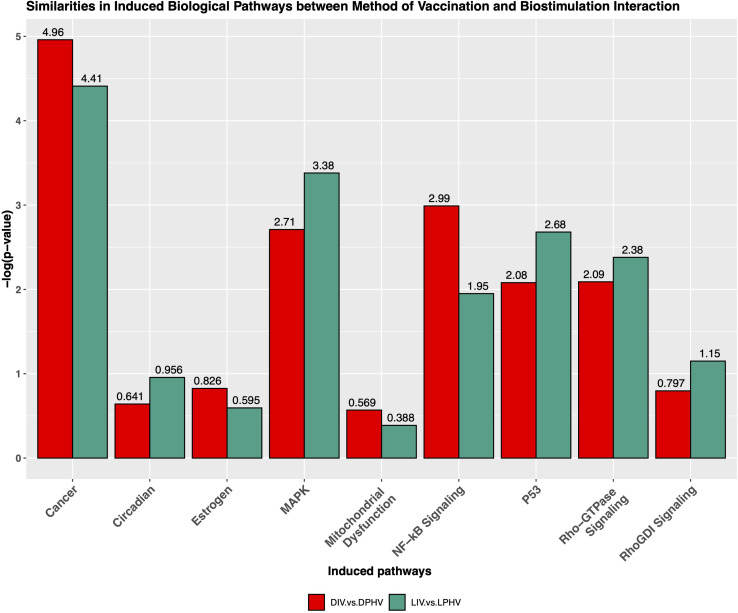
Similarities of non-canonical pathways induced by the interaction of the method of vaccination and green monochromatic light interaction.

The cardiac hypertrophy signaling pathway was predicted to be activated in both comparisons presented above, with Z-scores of 6.6 and 6.4 in DIV vs. DPHV and LIV vs. LPHV, respectively. Finding this as the top activated pathway in both comparisons shows that, perhaps unsurprisingly, cardiac hypertrophy was a crucial developmental process unaffected by lighting or vaccination status. However, it is valuable to know that lighting during incubation is not detrimental to normal developmental processes. Supplementary Table 7 shows genes involved in cardiac hypertrophy signaling (enhanced) canonical pathway. Furthermore, we saw that the non-canonical pathways, cancer, NF-κB signaling, mitochondrial dysfunction pathway, MAPK, Rho-GTPase signaling, P53, RhoGDI signaling, and circadian pathways were activated among LIV vs. LPHV and DIV vs. DPHV comparisons (Figure 8).

**FIGURE 9:**
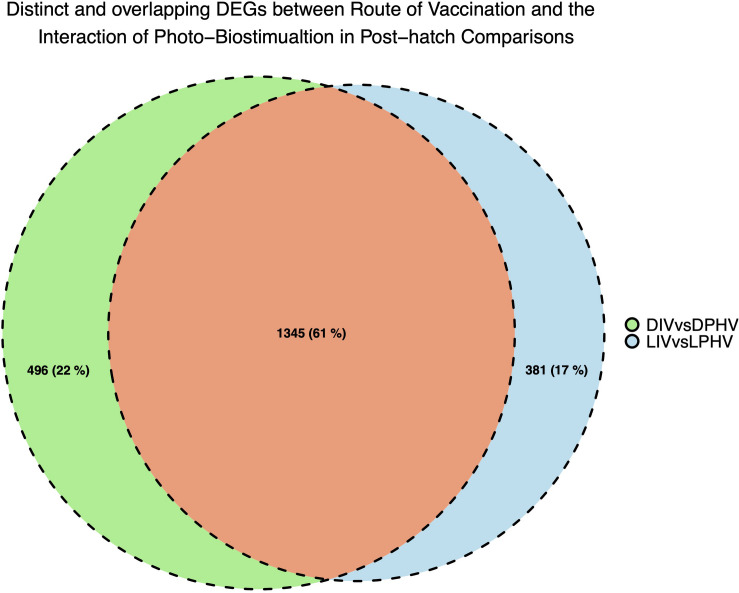
Unique and shared DEGs between method of vaccination and the interaction of green monochromatic light biostimulation in spleen tissues in post-hatch comparisons (DIV vs. DPHV and LIV vs. LPHV), stimulated by green monochromatic light during incubation.

We analyzed the shared and unique enhanced DEGs (LogFC > | 2|) between the DIV vs. DPHV and LIV vs. LPHV comparisons, to determine which expression differences are attributable to the light biostimulation alone. We found 381 genes unique to the LIV vs. LPHV group, after excluding those shared with DIV vs. DPHV (Figure 9). Of these, 327 genes (186 upregulated and 96 downregulated) were assigned to 36 canonical activated pathways in IPA, whereas four were predicted to be inhibited (Supplementary Table 6). The top five significant activated pathways were “Integrin Signaling,” “Reelin Signaling in Neurons,” “Amyotrophic Lateral Sclerosis Signaling,” “p53 Signaling,” and “ILK Signaling,” while the canonical pathways predicted to be inhibited are “T Cell Exhaustion Signaling Pathway,” “Protein Kinase A Signaling,” “Phospholipase C Signaling,” and “Endocannabinoid Cancer Inhibition Pathway.” On the other hand, the non-canonical pathways or induced pathways unique to the LIV vs. LPHV group showed enrichment of “P53,” “MAPK,” “Circadian,” and “Rho-GTPase Signaling” pathways. Two of the pathways (Integrin signaling, and ILK signaling pathways) are central to cell adhesion, and play a role in cell proliferation and differentiation, especially during development (Harburger and Calderwood, 2009; Tarekegn et al., 2020). Integrin signaling is a mediator of leukocyte migration and activation (Abram and Lowell, 2009), and therefore, this pathway may indicate a role in leukocyte signaling and function. As our data is generated with bulk RNAseq, we cannot determine if this activated pathway is informative about leukocytes specifically or broadly about the extracellular matrix of the solid tissue.

Ten networks emerged from the DEGs unique to the LIV vs. LPHV group that highlight the activities that are attributable to incubation with monochromatic green light (Supplementary Table 8). The networks are involved in biological functions classified into “Cell Morphology, Tissue Development, and Cell Maintenance,” “Cell-To-Cell Signaling and Interaction,”“Lipid Metabolism, Molecular Transport, and Small Molecule Biochemistry,” and “Cell-mediated Immune Response, Lymphoid Tissue Structure, and Development.” The top canonical pathway and the top induced pathway were integrin signaling and P53 pathways, respectively. As referenced above, integrins play an important role in cell-to-cell and cell-extracellular matrix (ECM) interactions, whereas p53 is a transcription factor and tumor suppressor. These classifications are supported by the network terms “Cellular Assembly and Organization,” “Cellular Function and Maintenance,” and “Tissue Development.” In summary, green monochromatic lighting during incubation enhanced cell signaling and cell proliferation or differentiation, following vaccination. If these enhanced activities translate to improved immune responses, it would suggest a synergistic effect of lighting on improved immune responses. As there were relatively few DEGs in comparison with light as the only factor, our results suggest a significant role for a small number of genes.

In summary, our results showed that the method of vaccination had a profound effect on gene expression in the post-hatch spleen, whereas the effect of green light was minimal in the post-hatch comparisons. This finding is not surprising given that all birds were on the same lighting treatments after hatching. There was a minor but notable interaction between *in ovo* lighting and vaccination on key immune developmental processes. This suggests that continuing lighting schemes in the post-hatch environment may be necessary to reinforce the pre-hatch activity patterns. Our study shows that the effects of *in ovo* lighting dissipate if the same lighting is not maintained in the post-hatch environment. Finally, our study emphasizes the need for continued investigation of *in ovo* lighting for stimulating responses to vaccinations.

## Data Availability Statement

The data presented in the study are deposited in the NCBI SRA repository, accession number PRJNA717366.

## Ethics Statement

The animal study was reviewed and approved by the Texas A&M University IACUC.

## Author Contributions

GSA and GA conceptualized the study. MI and JN performed the animal rearing and experiments. MI performed the molecular data generation and analyses. MI and GA performed the sequencing data analysis and interpretation. MI, JN, GSA, and GA wrote the manuscript. All authors contributed to the article and approved the submitted version.

## Conflict of Interest

The authors declare that the research was conducted in the absence of any commercial or financial relationships that could be construed as a potential conflict of interest.

## References

[B1] Abram C. L. Lowell C. A. (2009). The ins and outs of leukocyte integrin signaling. *Annu. Rev. Immunol.* 27 339–362. 10.1146/annurev.immunol.021908.132554 19302044 PMC3248397

[B2] Adam J. Dimond S. J. (1971). The effect of visual stimulation at different stages of embryonic development on approach behavior. *Anim. Behav.* 19 51–54. 10.1016/s0003-3472(71)80133-1 5149787

[B3] Amanna I. J. Slifka M. K. (2011). Contributions of humoral and cellular immunity to vaccine-induced protection in humans. *Virology* 411 206–215. 10.1016/j.virol.2010.12.016 21216425 PMC3238379

[B4] Anders S. Pyl P. T. Huber W. (2015). HTSeq — a Python framework to work with high-throughput sequencing data. *Bioinformatics* 31 166–169. 10.1093/bioinformatics/btu638 25260700 PMC4287950

[B5] Archer G. S. (2015). Timing of light exposure during incubation to improve hatchability, chick quality and post-hatch well-being in broiler chickens: 21 or 18 days. *Int. J. Poult. Sci.* 14 293–299. 10.3923/ijps.2015.293.299

[B6] Archer G. S. (2016). spectrum of white light during incubation: warm vs cool white LED lighting. *Int. J. Poult. Sci.* 15 343–348. 10.3923/ijps.2016.343.348

[B7] Archer G. S. (2017). Exposing broiler eggs to green, red and white light during incubation. *Animal* 11 1203–1209. 10.1017/S1751731117000143 28173889

[B8] Archer G. S. (2018). Effect of two different commercially available white light LED fixtures on broiler hatchability and chick quality. *Br. Poult. Sci.* 59 251–255. 10.1080/00071668.2018.1436160 29393660

[B9] Archer G. S. Jeffrey D. Tucker Z. (2017). Effect of the combination of white and red LED lighting during incubation on layer, broiler, and Pekin duck hatchability. *Poult. Sci.* 96 2670–2675. 10.3382/ps/pex040 28339779

[B10] Archer G. S. Mench J. A. (2017). Exposing avian embryos to light affects post-hatch anti-predator fear responses. *Appl. Anim. Behav. Sci.* 186 80–84. 10.1016/j.applanim.2016.10.014

[B11] Åvall K. Ali Y. Leibiger I. B. Leibiger B. Moede T. Paschen M. (2015). Apolipoprotein CIII links islet insulin resistance to β-cell failure in diabetes. *Proc. Natl. Acad. Sci. U.S.A.* 112 E2611–E2619. 10.1073/pnas.1423849112 25941406 PMC4443338

[B12] Bai X. Wang Y. Wang Z. Cao J. Dong Y. Chen Y. (2016). In ovo exposure to monochromatic lights affect posthatch muscle growth and satellite cell proliferation of chicks: role of IGF-1. *Growth Fact.* 34 107–118. 10.1080/08977194.2016.1199553 27362374

[B13] Bi R. Liu P. (2016). Sample size calculation while controlling false discovery rate for differential expression analysis with RNA-sequencing experiments. *BMC Bioinformatics* 17:146. 10.1186/s12859-016-0994-9 27029470 PMC4815167

[B14] Blackwell B. F. (2002). Understanding avian vision: the key to using light in bird management. *Vertebr._Pest_Conf.* 20:110129. 10.5070/V420110129

[B15] Bolger A. M. Lohse M. Usadel B. (2014). Trimmomatic: a flexible trimmer for Illumina sequence data. *Bioinformatics* 30 2114–2120. 10.1093/bioinformatics/btu170 24695404 PMC4103590

[B16] Cao J. Liu W. Wang Z. Xie D. Jia L. Chen Y. (2008). Green and blue monochromatic lights promote growth and development of broilers via stimulating testosterone secretion and myofiber growth. *J. Appl. Poult. Res.* 17 211–218. 10.3382/japr.2007-00043

[B17] Castell J. V. Gómez-Lechón M. J. David M. Andus T. Geiger T. Trullenque R. (1989). Interleukin-6 is the major regulator of acute phase protein synthesis in adult human hepatocytes. *FEBS Lett.* 242 237–239. 10.1016/0014-5793(89)80476-4 2464504

[B18] Chang H.-C. Guarente L. (2013). SIRT1 mediates central circadian control in the SCN by a mechanism that decays with aging. *Cell* 153 1448–1460. 10.1016/j.cell.2013.05.027 23791176 PMC3748806

[B19] Chiandetti C. Galliussi J. Andrew R. J. Vallortigara G. (2013). Early-light embryonic stimulation suggests a second route, via gene activation, to cerebral lateralization in vertebrates. *Sci. Rep.* 3:2701. 10.1038/srep02701 24048072 PMC3776965

[B20] Cooper J. B. (1972). Effect of light during incubation on hatchability of turkey eggs. *Poult. Sci.* 51 1105–1108. 10.3382/ps.0511105 4647573

[B21] Cray C. Zaias J. Altman N. H. (2009). Acute phase response in animals: a review. *Comp. Med.* 59 517–526. 20034426 PMC2798837

[B22] Csernus V. Becher P. Mess B. (1999). Wavelength dependency of light-induced changes in rhythmic melatonin secretion from chicken pineal gland in vitro. *Neuro Endocrinol. Lett.* 20 299–304. 11460088

[B23] de Freitas L. F. Hamblin M. R. (2016). Proposed mechanisms of photobiomodulation or low-level light therapy. *IEEE J. Sel. Top. Quantum Electron.* 22:7000417. 10.1109/JSTQE.2016.2561201 28070154 PMC5215870

[B24] Dimitrov K. M. Afonso C. L. Yu Q. Miller P. J. (2017). Newcastle disease vaccines-A solved problem or a continuous challenge? *Vet. Microbiol.* 206 126–136. 10.1016/j.vetmic.2016.12.019 28024856 PMC7131810

[B25] Dobin A. Davis C. A. Schlesinger F. Drenkow J. Zaleski C. Jha S. (2013). STAR: ultrafast universal RNA-seq aligner. *Bioinformatics* 29 15–21. 10.1093/bioinformatics/bts635 23104886 PMC3530905

[B26] Dobin A. Gingeras T. R. (2015). Mapping RNA-seq reads with STAR. *Curr. Protoc. Bioinformatics* 51 11.14.1–11.14.19. 10.1002/0471250953.bi1114s51 26334920 PMC4631051

[B27] Dolcet X. Llobet D. Pallares J. Matias-Guiu X. (2005). NF-kB in development and progression of human cancer. *Virchows Arch.* 446 475–482. 10.1007/s00428-005-1264-9 15856292

[B28] Duivenvoorden I. Teusink B. Rensen P. C. Romijn J. A. Havekes L. M. Voshol P. J. (2005). Apolipoprotein C3 deficiency results in diet-induced obesity and aggravated insulin resistance in mice. *Diabetes Metab. Res. Rev.* 54 664–671. 10.2337/diabetes.54.3.664 15734841

[B29] El-Gendy E. A. Abdelaziz M. M. Abdelfattah M. M. Badr Y. M. Salama M. S. (2015). Visible diode laser enhancement of exotic DNA uptake by fowl sperm. *J. Appl. Biol. Biotech.* 3 032–037. 10.7324/JABB.2015.3407

[B30] Ewels P. Magnusson M. Lundin S. Käller M. (2016). MultiQC: summarize analysis results for multiple tools and samples in a single report. *Bioinformatics* 32 3047–3048. 10.1093/bioinformatics/btw354 27312411 PMC5039924

[B31] Fairchild B. D. Christensen V. L. (2000). Photostimulation of turkey eggs accelerates hatching times without affecting hatchability, liver or heart growth, or glycogen content. *Poult. Sci.* 79 1627–1631. 10.1093/ps/79.11.1627 11092337

[B32] FAO OECD (2016). *OECD-FAO Agricultural Outlook 2016-2025.* Paris: OECD Publishing.

[B33] Foster R. G. Follett B. K. (1985). The involvement of a rhodopsin-like photopigment in the photoperiodic response of the Japanese quail. *J. Comp. Physiol.* 157 519–528. 10.1007/BF00615153

[B34] Gruys E. Toussaint M. J. M. Niewold T. A. Koopmans S. J. (2005). Acute phase reaction and acute phase proteins. *J. Zhejiang Univ. Sci. B* 6 1045–1056. 10.1631/jzus.2005.B1045 16252337 PMC1390650

[B35] Haas M. E. Attie A. D. Biddinger S. B. (2013). The regulation of ApoB metabolism by insulin. *Trends Endocrinol. Metab.* 24 391–397. 10.1016/j.tem.2013.04.001 23721961 PMC3810413

[B36] Halevy O. Piestun Y. Rozenboim I. Yablonka-Reuveni Z. (2006). In ovo exposure to monochromatic green light promotes skeletal muscle cell proliferation and affects myofiber growth in posthatch chicks. *Am. J. Physiol. Regul. Integr. Comp. Physiol.* 290 R1062–R1070. 10.1152/ajpregu.00378.2005 16269574

[B37] Harburger D. S. Calderwood D. A. (2009). Integrin signalling at a glance. *J. Cell Sci.* 122 159–163. 10.1242/jcs.018093 19118207 PMC2714413

[B38] Hieke A.-S. C. Hubert S. M. Athrey G. (2019). Circadian disruption and divergent microbiota acquisition under extended photoperiod regimens in chicken. *PeerJ* 7:e6592. 10.7717/peerj.6592 30886778 PMC6421066

[B39] Huth J. C. Archer G. S. (2015). Effects of LED lighting during incubation on layer and broiler hatchability, chick quality, stress susceptibility and post-hatch growth. *Poult. Sci.* 94 3052–3058. 10.3382/ps/pev298 26475072

[B40] Janciauskiene S. (2013). “Inflammation and acute phase proteins in haemostasis,” in *Acute Phase Proteins*, ed. Janciauskiene S. (London: InTech).

[B41] Jiang N. Wang Z. Cao J. Dong Y. Chen Y. (2016). Role of monochromatic light on daily variation of clock gene expression in the pineal gland of chick. *J. Photochem. Photobiol. B Biol.* 164 57–64. 10.1016/j.jphotobiol.2016.09.020 27643985

[B42] Jin E. Jia L. Li J. Yang G. Wang Z. Cao J. (2011). Effect of monochromatic light on melatonin secretion and arylalkylamine N-acetyltransferase mRNA expression in the retina and pineal gland of broilers. *Anat. Rec.* 294 1233–1241. 10.1002/ar.21408 21618440

[B43] Kanehisa M. Sato Y. Kawashima M. Furumichi M. Tanabe M. (2016). KEGG as a reference resource for gene and protein annotation. *Nucleic Acids Res.* 44 D457–D462. 10.1093/nar/gkv1070 26476454 PMC4702792

[B44] Kapczynski D. R. Afonso C. L. Miller P. J. (2013). Immune responses of poultry to Newcastle disease virus. *Dev. Comp. Immunol.* 41 447–453. 10.1016/j.dci.2013.04.012 23623955

[B45] Karu T. I. (1996). “Mechanisms of interaction of monochromatic visible light with cells,” in *Effects of Low-Power Light on Biological Systems SPIE Proceedings*, eds Karu T. I. Young A. R. (Bellingham: SPIE), 2–9.

[B46] Knott B. Berg M. L. Morgan E. R. Buchanan K. L. Bowmaker J. K. Bennett A. T. D. (2010). Avian retinal oil droplets: dietary manipulation of colour vision? *Proc. Biol. Sci.* 277 953–962. 10.1098/rspb.2009.1805 19939843 PMC2842729

[B47] Koj A. (1996). Initiation of acute phase response and synthesis of cytokines. *Biochim. Biophys. Acta* 1317 84–94. 10.1016/s0925-4439(96)00048-8 8950192

[B48] Krämer A. Green J. Pollard J. Tugendreich S. (2014). Causal analysis approaches in ingenuity pathway analysis. *Bioinformatics* 30 523–530. 10.1093/bioinformatics/btt703 24336805 PMC3928520

[B49] Lewis P. D. Morris T. R. (2000). Poultry and coloured light. *Worlds Poult. Sci. J.* 56 189–207. 10.1079/WPS20000015

[B50] Li J. Wang Z. Cao J. Dong Y. Chen Y. (2013). Melatonin receptor subtypes Mel1a and Mel1c but not Mel1b are associated with monochromatic light-induced B-lymphocyte proliferation in broilers. *Domest. Anim. Endocrinol.* 45 206–215. 10.1016/j.domaniend.2013.09.003 24209505

[B51] Liang S. C. Nickerson-Nutter C. Pittman D. D. Carrier Y. Goodwin D. G. Shields K. M. (2010). IL-22 induces an acute-phase response. *J. Immunol.* 185 5531–5538. 10.4049/jimmunol.0904091 20870942

[B52] Liu T. Zhang L. Joo D. Sun S.-C. (2017). NF-κB signaling in inflammation. *Signal Transduct. Target. Ther.* 2:17023. 10.1038/sigtrans.2017.23 29158945 PMC5661633

[B53] Liu W. Wang Z. Chen Y. (2010). Effects of monochromatic light on developmental changes in satellite cell population of pectoral muscle in broilers during early posthatch period. *Anat. Rec.* 293 1315–1324. 10.1002/ar.21174 20665810

[B54] Losi A. Gardner K. H. Möglich A. (2018). Blue-light receptors for optogenetics. *Chem. Rev.* 118 10659–10709. 10.1021/acs.chemrev.8b00163 29984995 PMC6500593

[B55] Lu B. Chen L. Liu L. Zhu Y. Wu C. Jiang J. (2011). T-cell-mediated tumor immune surveillance and expression of B7 co-inhibitory molecules in cancers of the upper gastrointestinal tract. *Immunol. Res.* 50 269–275. 10.1007/s12026-011-8227-9 21717068

[B56] Ma S. Wang Z. Cao J. Dong Y. Chen Y. (2018). Effect of monochromatic light on circadian rhythm of clock genes in chick pinealocytes. *Photochem. Photobiol.* 94 1263–1272. 10.1111/php.12963 29896808

[B57] Ma S. Wang Z. Cao J. Dong Y. Chen Y. (2019). BMAL1 but not CLOCK is associated with monochromatic green light-induced circadian rhythm of melatonin in chick pinealocytes. *Endocr. Connect.* 8 57–68. 10.1530/EC-18-0377 30533004 PMC6330720

[B58] Mano H. Fukada Y. (2007). A median third eye: pineal gland retraces evolution of vertebrate photoreceptive organs. *Photochem. Photobiol.* 83 11–18. 10.1562/2006-02-24-IR-813 16771606

[B59] Martin M. (2011). Cutadapt removes adapter sequences from high-throughput sequencing reads. *EMBnet J.* 17:10. 10.14806/ej.17.1.200

[B60] Masri S. Orozco-Solis R. Aguilar-Arnal L. Cervantes M. Sassone-Corsi P. (2015). Coupling circadian rhythms of metabolism and chromatin remodelling. *Diabetes Obes. Metab.* 17(Suppl. 1), 17–22. 10.1111/dom.12509 26332964 PMC4732882

[B61] McCarthy D. J. Chen Y. Smyth G. K. (2012). Differential expression analysis of multifactor RNA-Seq experiments with respect to biological variation. *Nucleic Acids Res.* 40 4288–4297. 10.1093/nar/gks042 22287627 PMC3378882

[B62] Mehner C. Radisky E. S. (2019). Bad tumors made worse: SPINK1. *Front. Cell Dev. Biol.* 7:10. 10.3389/fcell.2019.00010 30778387 PMC6369215

[B63] Nilsonne G. Lekander M. Åkerstedt T. Axelsson J. Ingre M. (2016). Diurnal variation of circulating interleukin-6 in humans: a meta-analysis. *PLoS One* 11:e0165799. 10.1371/journal.pone.0165799 27832117 PMC5104468

[B64] O’Brien M. (2012). The reciprocal relationship between inflammation and coagulation. *Top. Companion Anim. Med.* 27 46–52. 10.1053/j.tcam.2012.06.003 23031455

[B65] OECD (2018). “Meat,” in *Proceedings of the OECD-FAO Agricultural Outlook 2018-2027 OECD-FAO Agricultural Outlook*, (Paris: OECD), 149–162.

[B66] Parkar S. G. Kalsbeek A. Cheeseman J. F. (2019). Potential role for the gut microbiota in modulating host circadian rhythms and metabolic health. *Microorganisms* 7:41. 10.3390/microorganisms7020041 30709031 PMC6406615

[B67] Parvin R. Mushtaq M. M. H. Kim M. J. Choi H. C. (2014). Light emitting diode (LED) as a source of monochromatic light: a novel lighting approach for behaviour, physiology and welfare of poultry. *Worlds Poult. Sci. J.* 70 543–556. 10.1017/S0043933914000592

[B68] Raccoursier M. Thaxton Y. V. Christensen K. Aldridge D. J. Scanes C. G. (2019). Light intensity preferences of broiler chickens: implications for welfare. *Animal* 13 2857–2863. 10.1017/S175173111900123X 31134878

[B69] Robinson M. D. McCarthy D. J. Smyth G. K. (2010). edgeR: a Bioconductor package for differential expression analysis of digital gene expression data. *Bioinformatics* 26 139–140. 10.1093/bioinformatics/btp616 19910308 PMC2796818

[B70] Rozenboim I. Biran I. Chaiseha Y. Yahav S. Rosenstrauch A. Sklan D. (2004a). The effect of a green and blue monochromatic light combination on broiler growth and development. *Poult. Sci.* 83 842–845. 10.1093/ps/83.5.842 15141845

[B71] Rozenboim I. Piestun Y. Mobarkey N. Barak M. Hoyzman A. Halevy O. (2004b). Monochromatic light stimuli during embryogenesis enhance embryo development and posthatch growth. *Poult. Sci.* 83 1413–1419. 10.1093/ps/83.8.1413 15339018

[B72] Rozenboim I. Robinzon B. Rosenstrauch A. (1999). Effect of light source and regimen on growing broilers. *Br. Poult. Sci.* 40 452–457. 10.1080/00071669987197 10579401

[B73] Sadrzadeh A. Brujeni G. N. Livi M. Nazari M. J. Sharif M. T. Hassanpour H. (2011). Cellular immune response of infectious bursal disease and Newcastle disease vaccinations in broilers exposed to monochromatic lights. *Afr. J. Biotechnol.* 10 9528–9532.

[B74] Shafey T. M. (2004). Effect of lighted incubation on embryonic growth and hatchability performance of two strains of layer breeder eggs. *Br. Poult. Sci.* 45 223–229. 10.1080/00071660410001715821 15222419

[B75] Shafey T. M. Al-mohsen T. H. (2002). Embryonic growth, hatching time and hatchability performance of meat breeder eggs incubated under continuous green light. *Asian Austral. J. Anim. Sci.* 15 1702–1707. 10.5713/ajas.2002.1702

[B76] Shichida Y. Matsuyama T. (2009). Evolution of opsins and phototransduction. *Philos. Trans. R. Soc. Lond. B. Biol. Sci.* 364 2881–2895. 10.1098/rstb.2009.0051 19720651 PMC2781858

[B77] Shutze J. V. Lauber J. K. Kato M. Wilson W. O. (1962). Influence of incandescent and coloured light on chicken embryos during incubation. *Nature* 196 594–595. 10.1038/196594a0 13988825

[B78] Siegel P. B. Isakson S. T. Coleman F. N. Huffman B. J. (1969). Photoacceleration of development in chick embryos. *Comp. Biochem. Physiol.* 28 753–758. 10.1016/0010-406X(69)92108-2

[B79] Spengler M. L. Kuropatwinski K. K. Comas M. Gasparian A. V. Fedtsova N. Gleiberman A. S. (2012). Core circadian protein CLOCK is a positive regulator of NF-κB-mediated transcription. *Proc. Natl. Acad. Sci. U.S.A.* 109 E2457–E2465. 10.1073/pnas.1206274109 22895791 PMC3443185

[B80] Sultana S. Hassan M. R. Choe H. S. Ryu K. S. (2013). The effect of monochromatic and mixed LED light colour on the behaviour and fear responses of broiler chicken. *Avian Biol. Res.* 6 207–214. 10.3184/175815513X13739879772128

[B81] Tarekegn G. M. Khayatzadeh N. Liu B. Osama S. Haile A. Rischkowsky B. (2020). Ethiopian indigenous goats offer insights into past and recent demographic dynamics and local adaptation in sub-Saharan African goats. *Evol. Appl.* [Epub ahead of print]. 10.1111/eva.13118 PMC8287980 34295359

[B82] Tong Q. McGonnell I. M. Demmers T. G. M. Roulston N. Bergoug H. Romanini C. E. (2018). Effect of a photoperiodic green light programme during incubation on embryo development and hatch process. *Animal* 12 765–773. 10.1017/S1751731117002117 28835293

[B83] Venteclef N. Jakobsson T. Steffensen K. R. Treuter E. (2011). Metabolic nuclear receptor signaling and the inflammatory acute phase response. *Trends Endocrinol. Metab.* 22 333–343. 10.1016/j.tem.2011.04.004 21646028

[B84] Walter J. H. Voitle R. A. (1973). Effects of photoperiod during incubation on embryonic and post-embryonic development of quail and chickens. *Br. Poult. Sci.* 14 533–540. 10.1080/00071667308416062 4759985

[B85] Wang C. Shui K. Ma S. Lin S. Zhang Y. Wen B. (2020). Integrated omics in Drosophila uncover a circadian kinome. *Nat. Commun.* 11:2710. 10.1038/s41467-020-16514-z 32483184 PMC7264355

[B86] Withgott J. A. Y. (2000). Taking a bird’ s-eye view…in the UV. *Bioscience* 50:854.

[B87] Xie D. Wang Z. Cao J. Dong Y. Chen Y. (2008a). Effects of monochromatic light on proliferation response of splencyte in broilers. *Anat. Histol. Embryol.* 37 332–337. 10.1111/j.1439-0264.2008.00849.x 18294363

[B88] Xie D. Wang Z. X. Dong Y. L. Cao J. Wang J. F. Chen J. L. (2008b). Effects of monochromatic light on immune response of broilers. *Poult. Sci.* 87 1535–1539. 10.3382/ps.2007-00317 18648045

[B89] Yamao M. Araki M. Okano T. Fukada Y. Oishi T. (1999). Differentiation of pinopsin-immunoreactive cells in the developing quail pineal organ: an in-vivo and in-vitro immunohistochemical study. *Cell Tissue Res.* 296 667–671. 10.1007/s004410051326 10370152

[B90] Zhang L. Zhang H. J. Qiao X. Yue H. Y. Wu S. G. Yao J. H. (2012). Effect of monochromatic light stimuli during embryogenesis on muscular growth, chemical composition, and meat quality of breast muscle in male broilers. *Poult. Sci.* 91 1026–1031. 10.3382/ps.2011-01899 22399743

[B91] Zhang L. Zhang H. J. Wang J. Wu S. G. Qiao X. Yue H. Y. (2014). Stimulation with monochromatic green light during incubation alters satellite cell mitotic activity and gene expression in relation to embryonic and posthatch muscle growth of broiler chickens. *Animal* 8 86–93. 10.1017/S1751731113001882 24168791

[B92] Zhang L. Zhu X. D. Wang X. F. Li J. L. Gao F. Zhou G. H. (2016). Green light-emitting diodes light stimuli during incubation enhances posthatch growth without disrupting normal eye development of broiler embryos and hatchlings. *Asian Austral. J. Anim. Sci.* 29 1562–1568. 10.5713/ajas.15.0976 26954202 PMC5088375

[B93] Zhang Z. Cao J. Wang Z. Dong Y. Chen Y. (2014). Effect of a combination of green and blue monochromatic light on broiler immune response. *J. Photochem. Photobiol. B Biol.* 138 118–123. 10.1016/j.jphotobiol.2014.05.014 24927232

